# Cell Cytoskeleton and Stiffness Are Mechanical Indicators of Organotropism in Breast Cancer

**DOI:** 10.3390/biology10040259

**Published:** 2021-03-25

**Authors:** Kai Tang, Ying Xin, Keming Li, Xi Chen, Youhua Tan

**Affiliations:** 1 Shenzhen Research Institute, The Hong Kong Polytechnic University, Shenzhen 518000, China; kai-vincent.tang@connect.polyu.hk (K.T.); ying-xy.xin@connect.polyu.hk (Y.X.); keming.li.li@connect.polyu.hk (K.L.); xi.cc.chen@connect.polyu.hk (X.C.); 2 Department of Biomedical Engineering, The Hong Kong Polytechnic University, Hong Kong 999077, China

**Keywords:** cell stiffness, organotropism, cytoskeleton, cell mechanics, mechanoadaptation

## Abstract

**Simple Summary:**

Cancer cell dissemination exhibits organ preference or organotropism. Although the influence of intrinsic biochemical factors on organotropism has been intensely studied, little is known about the roles of mechanical properties of metastatic cancer cells. Our study suggests that there may be a correlation between cell cytoskeleton/stiffness and organotropism. We find that the cytoskeleton and stiffness of breast cancer cell subpopulations with different metastatic preference match the mechanics of the metastasized organs. The modification of cell cytoskeleton significantly influences the organotropism-related gene expression pattern and mechanoresponses on soft substrates which mimic brain tissue stiffness. These findings highlight the key role of cell cytoskeleton in specific organ metastasis, which may not only reflect but also impact the metastatic organ preference.

**Abstract:**

Tumor metastasis involves the dissemination of tumor cells from the primary lesion to other organs and the subsequent formation of secondary tumors, which leads to the majority of cancer-related deaths. Clinical findings show that cancer cell dissemination is not random but exhibits organ preference or organotropism. While intrinsic biochemical factors of cancer cells have been extensively studied in organotropism, much less is known about the role of cell cytoskeleton and mechanics. Herein, we demonstrate that cell cytoskeleton and mechanics are correlated with organotropism. The result of cell stiffness measurements shows that breast cancer cells with bone tropism are much stiffer with enhanced F-actin, while tumor cells with brain tropism are softer with lower F-actin than their parental cells. The difference in cellular stiffness matches the difference in the rigidity of their metastasized organs. Further, disrupting the cytoskeleton of breast cancer cells with bone tropism not only elevates the expressions of brain metastasis-related genes but also increases cell spreading and proliferation on soft substrates mimicking the stiffness of brain tissue. Stabilizing the cytoskeleton of cancer cells with brain tropism upregulates bone metastasis-related genes while reduces the mechanoadaptation ability on soft substrates. Taken together, these findings demonstrate that cell cytoskeleton and biophysical properties of breast cancer subpopulations correlate with their metastatic preference in terms of gene expression pattern and mechanoadaptation ability, implying the potential role of cell cytoskeleton in organotropism.

## 1. Introduction

Metastasis is the leading cause of cancer-related death [1]. Cancer cells do not randomly disseminate to and colonize other organs but rather have the preferred metastatic sites for each cancer, which is defined as organotropism. Different cancer types and subpopulations show different organotropisms. For example, prostate cancer tends to relapse in bones, while uveal melanoma typically metastasizes to the liver [2]. Breast cancer tends to metastasize distantly to the bone, brain, liver, lung, and distant lymph-nodes [3]. The most common breast cancer metastasis is bone metastases, which occurs in about 50% of metastatic breast cancer patients [4]. About 25% and 20% of breast cancer relapse is found in liver and lung and brain, respectively [4,5]. A variety of both intrinsic and extrinsic factors has been demonstrated to play important roles in the organotropism of cancer cells, including circulation patterns, intrinsic characteristics of cancer cells, organ specificity, ecological niche, and the microenvironment (ME) of tumor cells and the host [6,7]. However, the molecular mechanisms remain unclear. Therefore, understanding the underlying mechanisms of organotropic metastasis helps develop better diagnosis and/or treatment strategies and eventually improve the outcomes of cancer patients.

The influence of intrinsic biochemical factors on tumor organotropic metastasis has been intensely studied. Metastatic cancer cells derived from specific organs show preference to disseminate to the same site [5]. Intrinsic properties of cancer cells, such as organotropism gene signatures and pathways, are involved in organ-specific extravasation and colonization [8,9,10,11,12]. Dickkopf-1 secreted by breast cancer cells had distinct effects on lung metastasis and bone metastasis, which promoted bone metastasis through canonical Wnt signaling but inhibited lung metastasis via noncanonical Wnt signaling [13]. The COX2-MMP1/CCL7 axis enhances brain metastasis ability of breast cancer cells by promoting blood–brain barrier permeability and the growth of tumor-initiating cells [14]. It is well known that mechanical properties of cancer cells, as important intrinsic characteristics, are strongly correlated with their malignancy [15,16]. Malignant transformation driven by genetic mutations is accompanied by specific changes in cellular mechanical properties (such as stiffness and viscosity) [17]. Many previous studies show that the stiffness of tumor cells is lower than that of the corresponding normal cells. For example, normal breast epithelial cells become significantly softer after transformation [18,19]. The mechanical stiffness of tumor cells is highly heterogeneous [20] and significantly correlated with their malignant ability [15]. Cell softness is a unique mechanical signature of highly tumorigenic and metastatic tumor cells [21]. Low stiffness of tumor cells can help undergo extravasation during metastasis [22]. Softening tumor cells upregulates their self-renewal capacity [23,24]. Although the correlation between cell stiffness and tumor cell malignancy has been demonstrated, the relationship between cellular mechanical properties and metastatic preference remains unclear.

The mechanical property of a cell is mainly determined by its cytoskeleton and related proteins, including three major components: actin filaments, intermediate filaments, and microtubules [25]. Among them, actin filaments are believed to be one main contributor to cell stiffness [26]. A close correlation between actin filaments and cell stiffness has been well demonstrated by using disruptive pharmacological agents, such as cytochalasin D (Cyto D) [27]. In addition, it is well known that most adherent cells are under prestress state and exert contractile traction on substrates to sense substrate properties [28]. There are studies showing that cell contractility directly contributes to cell mechanics [29,30]. Myosin II binds to actin filaments and generates cellular contractility and its activity is regulated by myosin light-chain kinase (MLCK) and Rho-associated protein kinase (ROCK) [31]. Tumor cells with defective myosin bundles show an enhanced deformation ability compared to control cells [32,33]. Therefore, cellular stiffness can be effectively modulated by targeting cell cytoskeleton and contractility. However, very few studies investigate the role of actin cytoskeleton and cellular contractility in tumor organotropism.

In this study, the cytoskeleton and stiffness of various breast cancer cell subpopulations with distinct metastatic tropism were measured by immunofluorescent staining and atomic force microscopy. To explore the role of cell cytoskeleton in organotropism, the cytoskeleton was stabilized and disrupted in breast cancer cells with brain and bone tropism, respectively. The expressions of the genes specifically related to brain/bone metastasis were examined. The mechanosensitivity of these treated cells was tested on soft substrates that mimicked the stiffness of brain tissue, including cell spreading and proliferation.

## 2. Materials and Methods

### 2.1. Cell Culture

MDA-MB-231 (231 for short), MDA231-BrM2-831 (231-BrM for short), MDA-BoM-1833 (231-BoM for short), and MDA231-LM2-4175 (231-LM for short) cells were purchased from Memorial Sloan Kettering Cancer Center. All experiments were performed on parental 231 breast cancer cells, 231-BrM breast cancer cells that specifically metastasize to the brain, 231-BoM breast cancer cells that specifically metastasize to the bone, and 231-LM breast cancer cells that specifically metastasize to the lung tissue [8,9,10]. All cells were maintained in Dulbecco’s Modified Eagle Medium (DMEM) supplemented with 10% fetal bovine serum (FBS) and 1% penicillin/streptomycin in T25 flask at 37 °C and 5% CO_2_. All culture reagents were purchased from HyClone.

### 2.2. Pharmacologic Treatment

In brief, cells were seeded in a 6-well plate until ~70% of confluency reached. Cells were then treated with 2 or 6 μM Y-27632 (Selleck Chemicals), 2 or 6 μM blebbistatin (Sigma-Aldrich, St. Louis, MO, USA), 0.1 or 0.3 μM cytochalasin D (Tocris Bioscience, Manchester, UK), 5 or 50 nM narciclasine (Selleck Chemicals, Houston, TX, USA), 30 or 100 nM jasplakinolide (Selleck Chemicals, Houston, TX, USA) for 24 h without washing before total RNA extraction and F-actin staining. For the morphology measurement and proliferation assay, cells were pretreated with this drug for 24 h, which was washed away before harvesting the treated cells and reseeding them on tissue culture plates or polyacrylamide hydrogels for 24 h incubation. All the drugs were dissolved in DMSO to prepare the stock solution following the manufacturer’s instructions and then diluted with the full medium into working solution. DMSO was used as the vehicle to treat the control groups.

### 2.3. Polyacrylamide Hydrogels (PA gels) Preparation

Hydrogel manufacturing was performed as previously described [34,35]. Briefly, acrylamide and bis-acrylamide were mixed to their desired concentrations in distilled H_2_O. 1 % *v*/*v* ammonium persulfate (APS, Sigma-Aldrich, St. Louis, MO, USA), and 0.1% *v*/*v* methylethylenediamine (TEMED, Sigma-Aldrich, St. Louis, MO, USA) were added to gel solution and vortexed quickly. Twenty-five μL gel solution was quickly pipetted onto the chloro-silanated side of the glass slides, and the amino-silanated coverslips were covered. The gel was allowed to polymerize for 5 to 30 min. The PA gels were coated with 0.2 mg/mL rat-tail collagen type I (Sigma-Aldrich, St. Louis, MO, USA) via sulfosuccinimidyl 6-(4′-azido-2′-nitrophenylamino)hexanoate (sulfo-SANPAH; Sigma-Aldrich, St. Louis, MO, USA) crosslinker. Collagen-coated PA gels were sterilized with UV for 15 min and then soaked in the full medium for at least 30 min immediately prior to use.

### 2.4. AFM Measurements

Atomic force microscope (AFM, Bruker, Billerica, MA, USA) with silicon nitride cantilevers of spring constant k at 0.02 N/m (MLCT, Bruker, Billerica, MA, USA) was used to measured cell stiffness at room temperature [36]. The force F between tip and cell was the product of the cantilever deflection δ and k, i.e., F = k × δ. The cell Young’s modulus E was determined by fitting force-indentation curves with Sneddon’s modification of the Hertzian model for a pyramidal tip, i.e., F = 2/π × tan(α) × E/(1 − v^2^) × d^2^, where d was the indentation depth, α was the half tip angle, v was 0.5. In addition, d was kept within 500 nm at 1 Hz to avoid the possible effects of substrate and cell injury. Five force curves per cell were measured in the perinuclear region of cells (to avoid the potential influence from the substrate at the thin-cell periphery). The stiffness values were averaged for individual cells. An inverted microscope (Nikon, Tokyo, Japan) was combined with the AFM to be able to control tip and sample positioning.

### 2.5. Cell Morphology Analysis

Cells after treatment were seeded on tissue culture plates or polyacrylamide hydrogels for 24 h. Then brightfield images were captured by using an inverted microscope (Nikon, Tokyo, Japan) without fixation, from which cell boundary was traced using the software ImageJ (NIH, Bethesda, MD, USA). The parameters of cell morphology (area, circularity, and aspect ratio) were then analyzed using ImageJ as well.

### 2.6. Proliferation Assay

EdU stock solution (Beyotime, Shanghai, China) was added to the cell culture medium in 1:500 to prepare 2× working solution. After being preheated at 37 °C, the working solution was added to an equal volume of cell-culture medium, which was then used to incubate the cells for 2 h at 37 °C. These cells were detached, fixed, and permeabilized. Next, 0.5 mL of Click reaction reagent was added to stain the cells for 30 min in dark. The cells were stained with DAPI for 10 min. After washing, the percentage of EdU positive cells were analyzed by flow cytometry.

### 2.7. Quantitative RT-PCR Analysis

Total mRNAs were extracted by the E.Z.N.A.^®^ Total RNA Kit (Omega, Norcross, GA, USA), and complementary DNAs were synthesized using the RevertAid First Strand cDNA Synthesis Kit (Thermo, Waltham, MA, USA) following manufacturers’ recommended protocols, respectively. Quantitative RT-PCR was carried out using the Forget-Me-Not EvaGreen qPCR Master Mix with Rox (Biotium, San Francisco, CA, USA) and CFX96 Real-Time System (Bio-Rad, Hercules, CA, USA). All sequences of primers were designed using the National Centre for Biotechnology Information (NCBI, Bethesda, MD, USA) database and listed in Table S1. Relative gene expression was evaluated by using the ΔΔCT method and normalized to the expression of human glyceraldehyde 3-phosphate dehydrogenase (GAPDH).

### 2.8. F-actin Staining

CytoPainter F-actin labeling kit (Abcam, Cambridge, MA, USA) was used to stain F-actin. Briefly, cells were fixed with 4.0% formaldehyde in PBS at room temperature for 10–30 min and permeabilized with 0.1% Triton X-100 in PBS for 5 min. Next, cells were stained with 1× green fluorescent Phalloidin conjugate working solution at room temperature for 60 min. Cells were rinsed gently with PBS 2–3 times to remove excess dye and stained with DAPI before imaging by the inverted fluorescent microscope (Nikon) using FITC and DAPI channel, respectively. Fluorescence intensity was quantified using ImageJ (NIH).

### 2.9. Transfection

Cells were transfected with the appropriate quantity of small-interfering RNA (siRNA) using Lipofectamine 3000 (Life Technologies, Carlsbad, CA, USA) following the manufacturer’s recommended protocol. In brief, cells were seeded in a 6-well plate until 70–90% confluency before transfection. Lipofectamine 3000 reagent and siRNA were diluted in Opti-MEM medium, respectively. Then, the diluted siRNA was added into diluted lipofectamine 3000 reagent at 1:1 ratio and incubated for 10–15 min at room temperature. The siRNA-lipid complex was added to cells for 2 day before the assays. The siRNA specific for mDia1 (5′-AAAGGCAGAGCCACACUUCCU-3′) and control siRNA (5′-AUUGUAUGCGAUCGCAGAC-3′) were designed and synthesized by Hanbio Biotechnology.

### 2.10. Statistical Analysis

All the results were expressed as mean ± SEM in this study. At least three independent tests were used for each experiment. A two-tailed Student’s *t*-test was used to analyze the comparisons between the two groups. ANOVA analysis was used for the statistics among multiple group comparisons. The post hoc Tukey or Bonferroni test was adopted in the ANOVA analysis for the comparisons with equal or unequal sample sizes, respectively. The significance level was set at *p* < 0.05.

## 3. Results

### 3.1. Biophysical Properties of Breast Cancer Cells Subpopulations Are Correlated with Their Metastatic Preference

**Figure 1:**
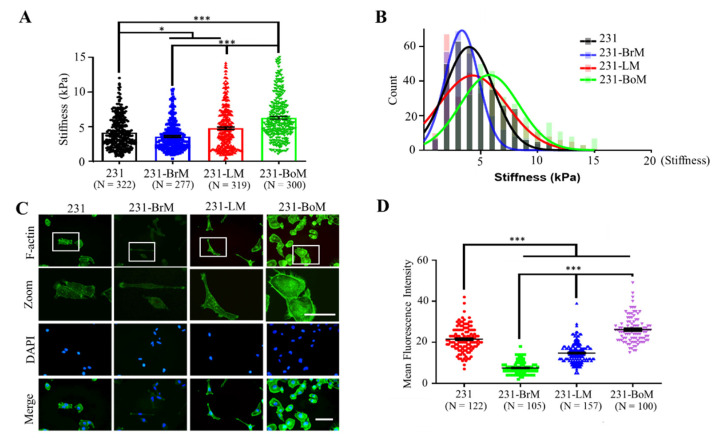
MDA-MB-231 (231) derivatives with the metastatic preference to brain (231-BrM), lung (231-LM), and bone (231-BoM) exhibit increasing F-actin and cellular stiffness. (**A**) Cellular stiffness of 231-BrM cells is lower than that of 231-LM cells that are softer than 231-BoM cells. Tumor cell stiffness was measured by atomic force microscopy. Three independent experiments. (**B**) The histogram of tumor cell stiffness in (**A**). The Young’s modulus distribution was fitted with Gaussian functions (lines). (**C**) The fluorescence imaging of F-actin in 231 derivatives with different metastatic preference. Tumor cells were stained with phalloidin (green) and DAPI (blue). The outlined regions in the top panel were enlarged in the second panel. The representative images were presented. (**D**) Quantification of the fluorescence intensity of F-actin in (**C**). Scale bar: 100 μm. Three independent experiments. The data represent mean ± SEM. * *p* < 0.05; and *** *p* < 0.001.

Cell mechanics are related to various cellular functions [37]. However, the relationship between mechanical properties of cancer cells and organotropism remains unclear. To address this question, we measured the stiffness of parental MDA-MB-231 cells (231) and the derivatives with the preference to metastasize to bone (MDA-BoM-1833, 231-BoM for short), lung (MDA231-LM2-4175, 231-LM for short), and brain (MDA231-BrM2-831, 231-BrM for short) by atomic force microscopy (AFM) [8,9,10]. The result shows that 231-BrM cells exhibited lower cellular stiffness than parental 231 cells (Figure 1A,B), while 231-LM and 231-BoM cells had higher stiffness. In addition, 231-BoM cells showed the highest cell stiffness among these groups. These experimental findings indicate that the stiffness of 231-BrM, 231-LM, and 231-BoM cells exhibits a progressive elevation, which is correlated with the increasing stiffness of their preferred metastatic organs (Figure 1A,B). As a key cytoskeletal element, actin filament network is crucial in the determination of cell stiffness [38]. Then, F-actin was measured by FITC-phalloidin immunofluorescence staining in breast cancer cells with different organotropism. Consistent with the finding of cell stiffness, 231-BrM cells had the lowest level of F-actin, whereas 231-BoM cells showed the highest level of F-actin (Figure 1C,D). There also was a significant increasing trend for F-actin levels from 231-BrM, 231-LM, to 231-BoM cells. All these data demonstrate that breast cancer cell subpopulations with different metastatic preference exhibit distinct biophysical properties and that cell cytoskeleton and softness may reflect the organotropism of breast cancer cells.

### 3.2. Cell Cytoskeleton Influences the Organotropism-Related Gene Expression Pattern

**Figure 2:**
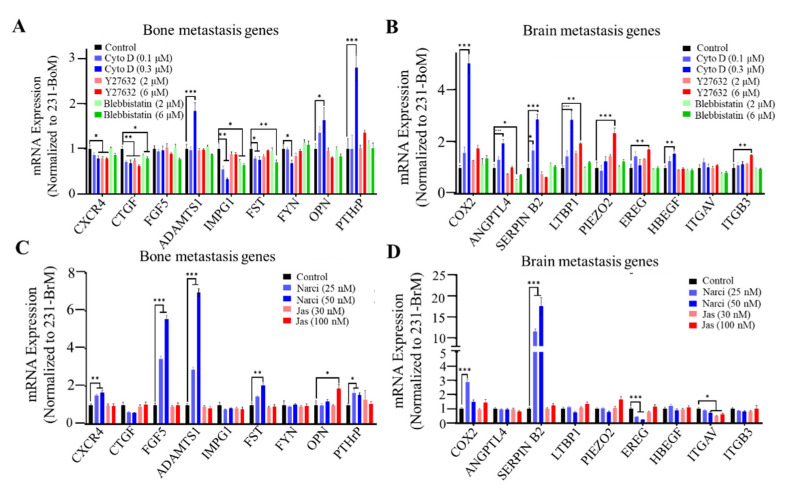
Cell cytoskeleton influences the expressions of bone/brain metastasis-related genes. (A, B) The effect of disrupting cell cytoskeleton on bone/brain metastasis-related genes in 231-BoM cells. The cytoskeleton of 231-BoM cells was disrupted by different dosages of Cyto D, Y27632, and Blebbistatin for 24 h. The expressions of bone metastasis-related genes (CXCR4, CTGF, FGF5, ADAMTS1, IMPG1, FST, FYN, OPN, and PTHrP) (**A**) and brain metastasis-related genes (COX2, ANGPTL4, Serpin B2, LTBP1, PIEZO2, EREG, HBEGF, ITGAV, and ITGB3) (**B**) were measured by quantitative RT-PCR. (C, D) The effect of stabilizing cell cytoskeleton on bone/brain metastasis-related genes in 231-BrM cells. The cytoskeleton of 231-BrM cells was stabilized by different dosages of Narci and Jas for 24 h. The expressions of bone metastasis-related genes (CXCR4, CTGF, FGF5, ADAMTS1, IMPG1, FST, FYN, OPN, and PTHrP) (**C**) and brain metastasis-related genes (COX2, ANGPTL4, Serpin B2, LTBP1, PIEZO2, EREG, HBEGF, ITGAV, and ITGB3) (**D**) were measured by quantitative RT-PCR. n= three independent experiments. The data represent mean ± SEM. * *p* < 0.05; ** *p* < 0.01; and *** *p* < 0.001.

We have demonstrated the correlation between cellular mechanical properties and metastatic organotropism. However, the role of cancer cell biophysical properties in metastatic tropism remains unclear. We hypothesized that cell cytoskeleton might influence the organotropism of breast cancer cells. To test this idea, the effect of cell cytoskeleton on the gene expression pattern related to brain and bone metastasis was investigated. F-actin inhibitor Cyto D, ROCK inhibitor Y27632, or myosin II inhibitor blebbistatin were used to disrupt the cytoskeleton of 231-BoM cells. On the other hand, F-actin polymerization activator jasplakinolide (Jas) and Rho activator narciclasine (Narci) were used to stabilize the cytoskeleton of 231-BrM cells. The expressions of the brain and bone metastasis gene signatures that have been identified previously were examined [8,9,10,14,39]. The data show that disrupting the cytoskeleton of 231-BoM cells using Cyto D or Y27632 had a minimal effect on bone metastasis-related genes beside a few exceptions (ADAMTS1, OPN, and PTHrP). In contrast, these pharmacologic treatments notably upregulated most brain metastasis-related genes in 231-BoM cells (COX2, ANGPTL4, SERPIN B2, LTBP1 PIEZO2, EREG, HBEGF, and ITGB3; Figure 2A,B). However, blebbistatin did not affect the expressions of both bone and brain metastasis-related genes. When 231-BrM cells were treated with Jas, the expressions of bone and brain metastasis-related genes were not obviously affected. In comparison, Narci treatment significantly enhanced five out of nine bone metastasis-related genes (CXCR4, FGF5, ADAMTS1, FST, and PTHrP; Figure 2C) and had minimal effects on the expressions of brain metastasis-related genes except the unexpected upregulation of COX2 and Serpin B2 (Figure 2D). To examine the efficacy of the pharmacologic perturbation, we measured the F-actin by FITC-phalloidin immunofluorescence staining after drug treatment. There was a significant decrease of F-actin level in 231-BoM cells treated with Cyto D. However, Y27632 and blebbistatin treatment did not decrease the F-actin level in 231-BoM cells and led to the minor effect on organotropism-related gene expression, which may be due to the low dosage of these drugs. Both the treatment of Jas and Narci enhanced the F-actin level in 231-BrM cells (Figure S1A–D). Our results indicate that pharmacologically disrupting/stabilizing the cytoskeleton of breast cancer cell subpopulations with bone/brain tropism influences the expressions of organotropism-related genes.

### 3.3. Cell Spreading and Proliferation of Breast Cancer Subpopulations with Distinct Metastatic Tropism Respond to Substrate Rigidity Dependent on Cell Cytoskeleton

**Figure 3:**
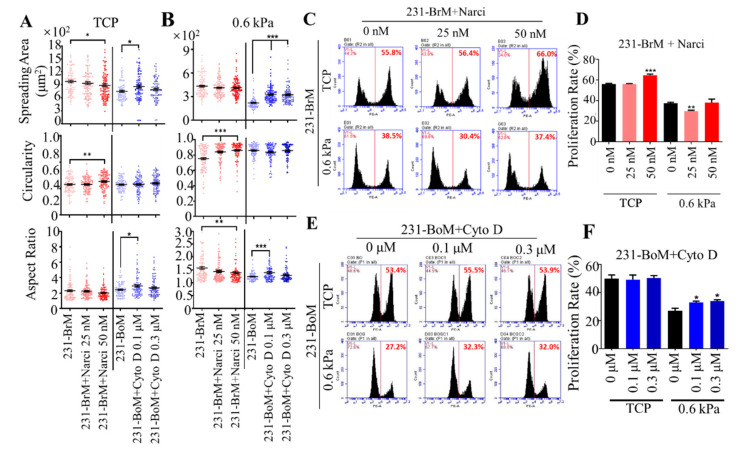
Stabilizing/disrupting the cytoskeleton of breast cancer cells with bone/brain tropism influences cell morphology and proliferation on soft substrates. (**A**,**B**) The influence of stabilizing and disrupting the cytoskeleton on the morphology of 231-BrM and 231-BoM cells. While 231-BrM cells were treated with Narci (25 nM, 50 nM), 231-BoM cells were treated with Cyto D (0.1 μM, 0.3 μM). Cell spreading area, circularity, and aspect ratio were then measured on TCP (**A**) and 0.6 kPa substrates (**B**). n > 100 cells/condition; 3 independent experiments. (**C**,**D**) Stabilizing the cytoskeleton of 231-BrM cells suppresses their proliferation on soft but not stiff substrates. In addition, 231-BrM cells were treated with 25 or 50 nM Narci and then cultured on TCP and 0.6 kPa substrates for 24 h, respectively. Then, cell proliferation was measured by EdU proliferation assay. n = 3. (**E**,**F**) Disrupting the cytoskeleton of 231-BoM cells enhances cell proliferation on soft but not stiff substrates. In addition, 231-BoM cells were treated with 0.1 or 0.3 μM Cyto D and then cultured on TCP and 0.6 kPa substrates for 24 h, respectively. n = 3. The data represent mean ± SEM. * *p* < 0.05; ** *p* < 0.01; and *** *p* < 0.001.

Our results have shown that cell cytoskeleton impacts the expressions of gene signatures related to organotropism. We next explored the influence of cell cytoskeleton on the response of breast cancer subpopulations with different organotropism to substrate rigidity. Notably, 231-BrM, 231-LM, and 231-BoM cells specifically metastasize to brain (0.1–1.0 kPa), lung (0.44 to 7.5 kPa), and bone (25–40 kPa) with distinct tissue stiffness [34,40]. To this end, the cytoskeleton of 231-BrM cells was stabilized by Narci, while the cytoskeleton 231-BoM cells was disrupted by Cyto D. These treated cancer cells were then cultured on tissue culture plates (TCPs) or polyacrylamide hydrogels with the stiffness of 0.6 kPa, which mimicked the mechanical stiffness of brain tissue. Cell spreading and morphology are positively correlated with cell proliferation and motility and thus can be used as effective indicators to determine whether the microenvironment is supportive of tumor cells [41,42]. The results show that when 231-BrM cells were treated with Narci, the circularity/aspect ratio was increased/decreased on soft substrates, although the spreading area was not affected. In contrast, disrupting the cytoskeleton of 231-BoM cells enhanced cell spreading area, aspect ratio but not circularity on both TCPs and soft substrates (Figure 3A,B). Cell proliferation is essential for disseminated tumor cells to develop into secondary tumors upon the arrival at distant organs. We found that 25 nM Narci treatment decreased cell proliferation rate on soft substrates but not on TCP. 50 nM Narci treatment increased cell proliferation on TCP, whereas this effect was abolished on soft substrates (Figure 3C,D). Modulating the cytoskeleton of 231-BoM cells with Cyto D had no obvious effect on their proliferation on TCPs. In contrast, inhibiting the cytoskeleton of 231-BoM cells increased their proliferation on soft substrates (Figure 3E,F). Taken together, these findings demonstrate that cell cytoskeleton may be a novel regulator of mechanical preference of breast cancer cells with distinct organotropism on soft substrates.

### 3.4. Silencing mDia 1 Enhances the Characteristics of Brain Metastasis in 231-BoM Cells

**Figure 4:**
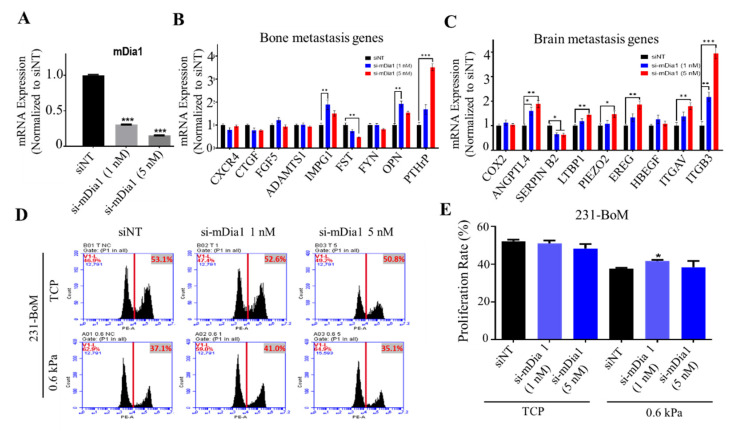
Silencing mDia1 in 231-BoM cells influences the expression profiles of the genes related to bone and brain metastasis and enhances cell proliferation on soft substrates. (**A**) The knockdown efficiency of mDia1. n = 3. (**B**) The influence of silencing mDia1 on the expressions of bone metastasis-related genes. n = 3. (**C**) The influence of silencing mDia1 on the expressions of brain metastasis-related genes. n=3. In addition, 231-BoM cells were transfected with mDia1 siRNAs, and the gene expression was examined in (**B**,**C**) by quantitative RT-PCR. (**D**,**E**) Silencing mDia1 promotes cell proliferation on soft but not stiff substrates. In addition, 231-BoM cells with mDia1 knockdown were cultured on TCP and 0.6 kPa substrates and cell proliferation was measured by EdU assay. n=3. The data represent mean ± SEM. * *p* < 0.05; ** *p* < 0.01; and *** *p* < 0.001.

mDia1, a downstream effector of RhoA, is a key driver for actin polymerization, which contributes to cell mechanics [43,44]. Previous studies show that the Piezo2-RhoA-mDia pathway is necessary for the homeostatic regulation of actin cytoskeleton and force transduction in 231-BrM cells [39]. To further explore the role of cell cytoskeleton and mechanics, 231-BoM cells were transfected with mDia1 siRNAs (Figure 4A) and their gene expression and cell proliferation were examined. We found that silencing mDia1 in 231-BoM cells did not alter the expressions of bone metastasis genes consistently, where three out of nine bone metastasis genes were upregulated (IMPG1, OPN, and PTHrP), while FST was downregulated (Figure 4B). In contrast, inhibiting mDia1 upregulated seven out of nine brain metastasis genes remarkably in 231-BoM cells (ANGPTL4, LTBP1, PIEZO2, EREG, ITGAV, ITGB3; Figure 4C). Furthermore, knocking down mDia1 at low dose (1 nM) promoted cell proliferation on soft substrates but not on TCPs (Figure 4D,E). These data suggest that silencing mDia1 enhances the expressions of brain metastasis genes and cell proliferation on soft substrates in 231-BoM cells, which indicate the increase of their brain metastasis ability.

## 4. Discussion

Many cancers show an organ-specific pattern of metastases. This phenomenon was first explained by the “seed and soil” theory proposed by Steven Paget [45], in which “seed” refers to cancer cells with the ability to metastasize, while “soil” refers to the organs with the supportive microenvironment. When the “seed” and “soil” fit each other, it is then possible for the organ-specific metastases to be developed. Both cancer cells and tumor microenvironment determine the inefficiency of the metastasis process [6,7]. In cancer cells, the key factors include the gene signature, stemness, tumor dormancy state, and tumor-secreted factors that play pivotal roles in mediating organ-specific metastasis [46]. Except for these biochemical mechanisms, mechanical cues play important roles in tumor metastasis [47], including tumor cell mechanics. It is well accepted that mechanical properties of tumor cells are linked to the invasive potential in pancreatic, ovarian, and breast cancer [15,48,49]. Highly invasive and metastatic cancer cells are more deformable than their less invasive counterparts, which may help cancer cells transit through narrow constrictions during the metastasis process. Our previous studies show that the reduced cytoskeleton and thus cell mechanics enhance the survival of circulating tumor cells under fluid shear force in the circulation system and promote their chemoresistance ability [50,51]. However, the role of tumor cell cytoskeleton and mechanics in organotropism remains unclear. The current study establishes the correlation between cancer cell cytoskeleton and organotropism. Breast cancer cell subpopulations with different organ preference have distinct F-actin and cell stiffness that match the stiffness of their targeted organs. Importantly, disrupting the cytoskeleton of breast cancer cell subpopulations with bone tropism upregulates brain metastasis-related genes and promotes their spreading and proliferation on soft substrates. On the other hand, stabilizing the cytoskeleton of breast cancer cell subpopulations with brain tropism upregulates bone metastasis-related genes and suppresses the spreading and proliferation on soft substrates. These findings suggest that cell cytoskeleton may not only correlate with but also influence organotropism and that cell cytoskeleton that matches the organ mechanics may enhance the ability of tumor cells to adapt to the metastasized organ, which may unveil a new role of cell cytoskeleton in tumor metastasis. Nevertheless, our results show that the Cyto D treatment could upregulate several bone metastasis-related genes, including ADAMTS1, OPN, and PTHrP, while silencing mDia1 increases the expressions of OPN and PTHrP. On the other hand, Narci and Jas treatment could enhance the expression of brain metastasis-related gene SERPIN B2 in 231-BrM cells. The unexpected changes of gene expression after the modulation in cell cytoskeleton may be due to the fact that the pharmacological treatment and genetic modification not only lead to the change in cell cytoskeleton but also mediate a series of alterations in their downstream signaling and cytoskeletal reorganization.

It is worthy to note that cell cytoskeleton and stiffness can be influenced by various extracellular signals [52]. Extracellular vesicles (EVs) secreted by cancer-associated fibroblasts can influence the mechanical state of cancer cells [53]. Mechanical microenvironments can also provide various cues that enable a cell to regulate its cytoskeleton and alter its mechanical state [52]. Many studies have shown that cells adapt their stiffness to match the compliance of their substrates through cytoskeletal reorganization [35,54]. In line with this notion, various degrees of stiffness of tumor cells within the same tumor tissue may be due to the heterogeneity of the tumor mechanical microenvironment [20]. The MDA-MB-231 derivatives were established through collecting metastatic cells from the secondary tumors in brain, lung, and bone with distinct tissue mechanics. Thus, it raises a question whether the unique cell cytoskeleton and stiffness of these derivatives are due to their adaption to the special mechanical microenvironment of the targeted organ or due to their intrinsic features regardless of extracellular factors. Furthermore, these metastatic cells have been cultured on TCPs for a long time after cell extraction, which may impact the cytoskeleton and stiffness of the corresponding primary metastatic cells. These important issues need to be rigorously investigated in the future.

The cytoskeleton provides the essential structure to transduce the external mechanical cues from the cell membrane to the nucleus, which regulates various cell functions [55]. Actin polymerization and actomyosin contractility synergistically regulate cell morphology, motility, division, and protein secretion [56]. Our previous results demonstrate that modulating cell cytoskeleton and contractility influences the expressions of the genes related to survival and drug resistance and regulates the apoptosis of circulating tumor cells under shear flow and the chemoresistance ability [50]. In this study, when 231-BrM cells were treated with Narci, the circularity/aspect ratio was increased/decreased on soft substrates, although spreading area was not affected. In contrast, disrupting the cytoskeleton of 231-BoM cells enhanced cell spreading area, aspect ratio but not circularity on both TCPs and soft substrates. Cytoskeleton-mediated mechanical signals have been linked closely to cell proliferation [57]. Our data show that targeting actin network might have distinct effect on tumor cells on substrates with different stiffness, as 50 nM Narci treatment increased cell proliferation on TCP, whereas this effect was abolished on soft substrates. Cyto D enhanced proliferation of 231-BoM cells on soft substrates but not TCP. Note that although the brain tissue is mechanically heterogeneous, the stiffness of the brain regions where tumor cell may infiltrate is below 1kPa, including cortex, cerebellum, and corpus callosum [58,59,60]. Therefore, it is reasonable to utilize 0.6 kPa-substrate to represent the mechanical microenvironment of the brain tissue and investigate the influence on the fate of tumor cells at the early stage of brain metastasis.

It is well known that cytoskeleton and actomyosin-mediated contractility contribute to cell mechanical stiffness. Disrupting the cytoskeleton or inhibiting myosin activity reduces cell stiffness, while stabilizing cytoskeleton or activating myosin activity increases mechanical stiffness [29,30,61,62]. To examine the role of cell cytoskeleton in organotropism, several pharmacological inhibitors that interfere the dynamics of actin cytoskeleton and cellular contractility are used in our study. Cyto D can inhibit actin polymerization by blocking the fast-growing barbed ends of actin filaments. Jas binds actin filaments and suppresses their disassembly. Narci triggers actin stress fiber formation by activating small GTPase RhoA. Blebbistatin directly inhibits myosin II activity to reduce cytoskeletal contractility, whereas Y27632 inhibits myosin II activator ROCK. Therefore, these drugs influence cell cytoskeleton and stiffness through different mechanisms. Consistently, our results show that Cyto D treatment but not the low dose of blebbistatin and Y-27632 significantly reduces F-actin amount, while Jas and Narci enhance the F-actin level. We find that Cyto D affects gene expression significantly, whereas blebbistatin and Y-27632 show minor effect on gene expression pattern in 231-BoM cells. This phenomenon may be due to the distinct working mechanisms of these pharmacologic treatments and low dosage of the drugs used in our study, since the adopted dose of Cyto D but not Y-27632/blebbistatin significantly reduces the F-actin amount (Figure S1). Moreover, previous studies show that cytoskeleton influences the differentiation of human pluripotent stem cells toward pancreatic β cells. However, the authors found that not all the drugs targeting cytoskeleton could induce endocrine differentiation [63]. This discrepancy is likely due to the different mechanistic effects of these compounds on the cytoskeleton [64]. Therefore, this effect cannot be excluded in the current study and may possibly contribute to the distinct influence of different pharmacologic treatments on the gene expression.

The response of tumor cells to mechanical cues is crucial for their outgrowth in the metastasized organs and depends on both intracellular structure and mechanical properties. Moreover, the cytoskeleton and stiffness of a cell should match the microenvironmental mechanics to sense and respond to the surrounding mechanical cues properly [65,66,67], suggesting that it is likely for soft/stiff tumor cells to survive and grow well in a soft/stiff niche. This idea is supported by the finding that cloning and multiplying breast cancer cells that are prone to bone and lung metastasis on the substrates with corresponding stiffness can significantly improve cell proliferation and migration [68]. Our study has investigated the role of cell cytoskeleton in the response of tumor cells with different metastatic tropism to the soft matrix. Stabilizing the cytoskeleton of 231-BrM cells decreases cell spreading and proliferation on soft substrates, while disrupting the cytoskeleton of 231-BoM cells enhances the spreading and proliferation. These data suggest that the deregulation of cell cytoskeleton may confer cells the outgrowth advantages in soft brain tissue, which should be further investigated. Although gene expression and mechanoadaptation ability to substrate rigidity can indicate organotropism to some extent, the direct evidence should be provided in the future to demonstrate the effect of cell cytoskeleton and stiffness on organ tropism, especially the test of organotropism using animal models after intracardiac injection of tumor cells with modulated cell cytoskeleton and mechanics.

## 5. Conclusions

In summary, this study suggests that there may be a correlation between cell cytoskeleton/stiffness and organotropism. The stiffness of breast cancer cell subpopulations with different metastatic preference matches the mechanics of the metastasized organs. Cell cytoskeleton significantly influences the organotropism-related gene expression pattern and mechanoresponses to soft substrates which mimic brain tissue stiffness. These findings highlight the key role of cell cytoskeleton in specific organ metastasis, which may not only reflect but also impact the metastatic organ preference.

## Data Availability

No new data were created or analyzed in this study. Data sharing is not applicable to this article.

## Supplementary Materials

The following are available online at https://www.mdpi.com/2079-7737/10/4/259/s1, Figure S1: F-actin levels for 231-BoM and 231-BrM cells after pharmacologic treatment. Table S1: List of primers.
