# Supplementary material for: Cell Cytoskeleton and Stiffness Are Mechanical Indicators of Organotropism in Breast Cancer
Source: Biology (Basel). 2021 Mar 25;10(4):259. doi: 10.3390/biology10040259 (PMC8064360; doi:10.3390/biology10040259)
Supplement: Supplementary file 1 [file biology-10-00259-s001.pdf]

Supplementary figure

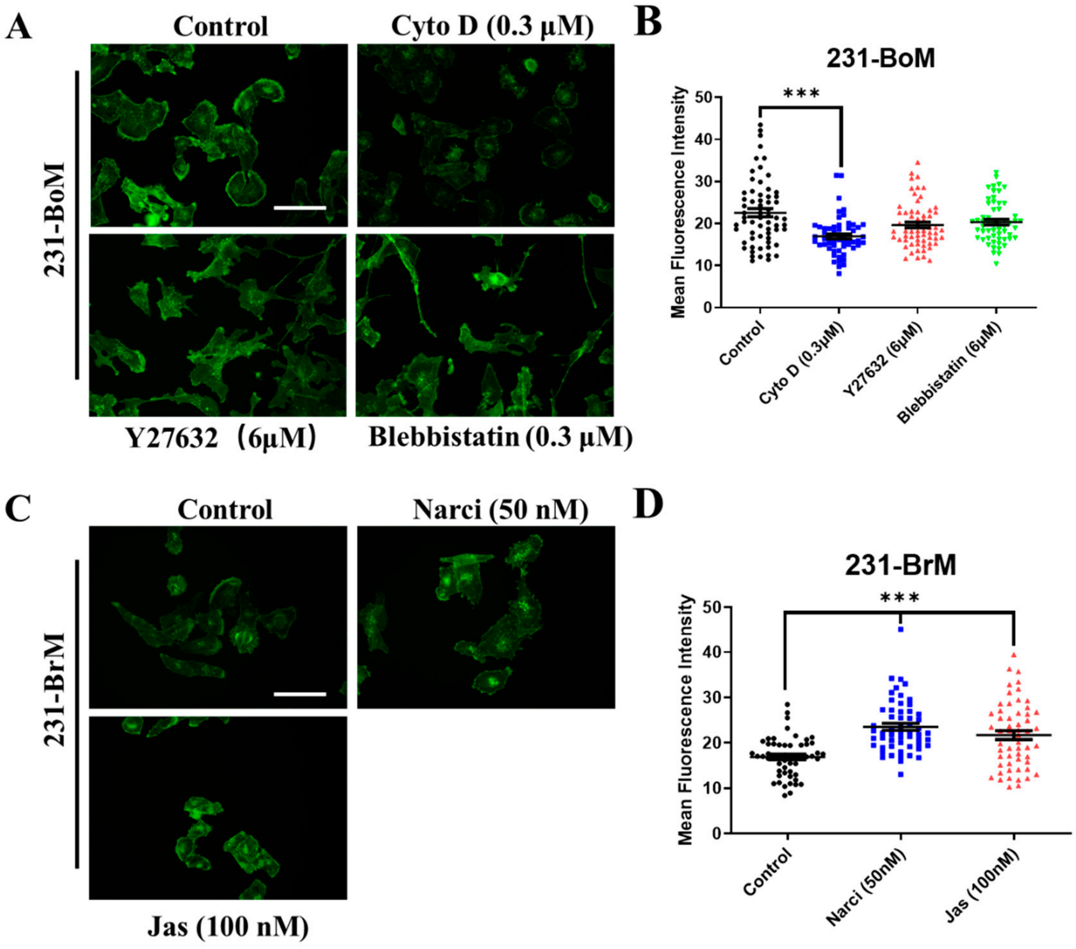

Figure S1. F-actin levels for 231-BoM and 231-BrM cell after pharmacologic treatment. (A) 231-BoM cells were treated with Cyto D (0.3μM), Y27632 (6μM), and blebbistatin (6μM) for 24h. The fluorescence images of F-actin were captured and the fluorescent intensity was quantified in (B). (C) 231-BrM cells were treated with Narci (50nM)and Jas (6μM) for 24h. The fluorescence images of F-actin were captured and the fluorescent intensity was quantified in (D). n>50; 3 independent experiments. The data represent mean ± SEM. Scale bar: 100μm. ***, p<0.001.

Supplementary table

Table S1: List of primers

Genes		Quantitative RT-PCR
CXCR4	5′ primer	GGTAGCGGTCCAGACTGATGA
	3′ primer	CCTATGCAAGGCAGTCCATGT
CTGF	5′ primer	CTCCTGCAGGCTAGAGAAGC
	3′ primer	GATGCACTTTTTGCCCTTCTT
FGF5	5′ primer	CCCGGATGGCAAAGTCAATGG
	3′ primer	TTCAGGGCAACATACCACTCCCG
ADAMTS1	5′ primer	TCCGTCATAGAAGATGATGGTTT
	3′ primer	GCATGTTAAACACGTGGCCTA
IMPG1	5′ primer	GGCTGTAGTCCTGCCAGAAG
	3′ primer	GTTGAGGCCTGATGAGTGGT
FST	5′ primer	ACCTGAGAAAGGCTACCTG
	3′ primer	ACTGAACCTGACCGTACACAACCTTGAAATCCCATAAA
FYN	5′ primer	AAGAGCCCCAGAAATTCACA
	3′ primer	CGCCAACGATCACAAACTT
OPN	5′ primer	TCCAACGAAAGCCATGACCA
	3′ primer	CTGTGGGGACAACTGGAGTG
PTHrP	5′ primer	CTCGGTGGAGGGTCTCAG
	3′ primer	TGGATGGACTTCCCCTTGT
COX2	5′ primer	TTCAACACACTCTATCACTGGC
	3′ primer	AGAAGCGTTTGCGGTACTCAT
ANGPTL4	5′ primer	TCCGTACCCTTCTCCACTTG
	3′ primer	AGTACTGGCCGTTGAGGTTG
SERPIN B2	5′ primer	GTTCATGCAGCAGATCCAGA
	3′ primer	CGCAGACTTCTCACCAAACA
LTBP1	5′ primer	CTTCCCCTGCCCGGTCT
	3′ primer	CTGCATCTTTATAGTTCTCACCACCA
PIEZO2	5′ primer	GACGGACACAACTTTGAGCCTG
	3′ primer	CTGGCTTTGTTGGGCACTCATTG
EREG	5′ primer	CTGCCTGGGTTTCCATCTTCT
	3′ primer	GCCATTCATGTCAGAGCTACACT
HBEGF	5′ primer	GGACCCATGTCTTCGGAAAT
	3′ primer	CCCATGACACCTCTCTCCAT
ITGAV	5′ primer	CTCGGGACTCCTGCTACCTC
	3′ primer	AAGAAACATCCGGGAAGACG
ITGB3	5′ primer	CCGTGACGAGATTGAGTCA
	3′ primer	AGGATGGACTTTCCACTAGAA
GAPDH	5′ primer	GCGACACCCACTCCTCCACCTTT
	3′ primer	TGCTGTAGCCAAATTCGTTGTCATA
